# A collection of read depth profiles at structural variant breakpoints

**DOI:** 10.1038/s41597-023-02076-4

**Published:** 2023-04-06

**Authors:** Igor Bezdvornykh, Nikolay Cherkasov, Alexander Kanapin, Anastasia Samsonova

**Affiliations:** Institute of Translational Biomedicine, Saint Petersburg State University, Saint Petersburg, 199004 Russia

**Keywords:** Software, Genetic databases, Data processing

## Abstract

SWaveform, a newly created open genome-wide resource for read depth signal in the vicinity of structural variant (SV) breakpoints, aims to boost development of computational tools and algorithms for discovery of genomic rearrangement events from sequencing data. SVs are a dominant force shaping genomes and substantially contributing to genetic diversity. Still, there are challenges in reliable and efficient genotyping of SVs from whole genome sequencing data, thus delaying translation into clinical applications and wasting valuable resources. SWaveform includes a database containing ~7 M of read depth profiles at SV breakpoints extracted from 911 sequencing samples generated by the Human Genome Diversity Project, generalised patterns of the signal at breakpoints, an interface for navigation and download, as well as a toolbox for local deployment with user’s data. The dataset can be of immense value to bioinformatics and engineering communities as it empowers smooth application of intelligent signal processing and machine learning techniques for discovery of genomic rearrangement events and thus opens the floodgates for development of innovative algorithms and software.

## Background & Summary

Structural variants are genomic alterations that encompass at least 50 nucleotides^1^. The term refers to a variety of events which include deletions, duplications, insertions, inversions, translocations and more complex rearrangements usually associated with mobile genetic elements^2^. Furthermore, SVs that change the number of copies of a DNA sequence are often defined as “copy number variants” (i.e., CNVs). Typically, SVs are single events, however in certain situations frequently occurring in cancer they may pile up resulting in large, complex, entangled combinations of alterations also known as chromosome shattering or chromothripsis^3,4^. Genome structural variation is a potent source of genetic diversity and may have a profound effect upon human health, as SVs are implicated in both germline and somatic disease ranging from developmental and neurological disorders to a wide spectrum of cancers^2,5–9^. SVs hold a great potential as molecular biomarkers to guide precision medicine^10–13^.

Robust and reproducible structural variation discovery still poses significant computational and algorithmic challenges^14,15^. Although, we are getting near to resolving structural variation in personal genomes with accuracy required for translational research^5,16,17^, faultless detection of SVs in many cases (e.g., insertions, CNV gains)^18^ remains notoriously difficult. Recent advances in technology, such as, long-read sequencing provide plenty of good reasons for cautious optimism on reaching a reasonable accuracy of SV discovery^19–23^. Nevertheless, the high cost and the low throughput of this strategy currently limits its general use. The short-read sequencing routinely used in a clinical setting and in nation-wide medical genetics initiatives makes the discovery, genotyping and characterisation of the variants difficult. SV discovery algorithms designed to process short sequencing fragments rely on uniformity and evenness of sequencing coverage profile (i.e., number of reads aligned to a genomic region or nucleotide), as well as read depth information for accurate detection of structural variants^18^. However, as sequencing coverage signal is discontinuous, heterogeneous, and irregular, often even erratic existing SV detection tools still generate highly discordant results^24–26^.

Over the course of the past decade SV discovery algorithms have generally explored two major strategies for variant detection, namely they either exploit read depth variability or base their discovery strategy on analysis of discordant alignment features. At present no single computational algorithm can detect SVs of all types and sizes in a robust, reliable manner. Moreover, as a rule, an approach which combines calls generated by several detection methods is required to achieve satisfactory performance^24,27–31^.

In this context, approaches exploring properties of depth of coverage (DOC) signal hold a tremendous potential, especially as a) relevant methodologies are applicable to data produced with both short- and long-read sequencing protocols, and b) it should be sufficient for discovery of the majority of SV classes regardless of their size and breakpoint location, as long as they distort the signal. The design of such tools calls for development of open access resources that aggregate and integrate signal coverage profiles in the vicinity of SV breakpoints, which so far is not available.

**Fig. 1:**
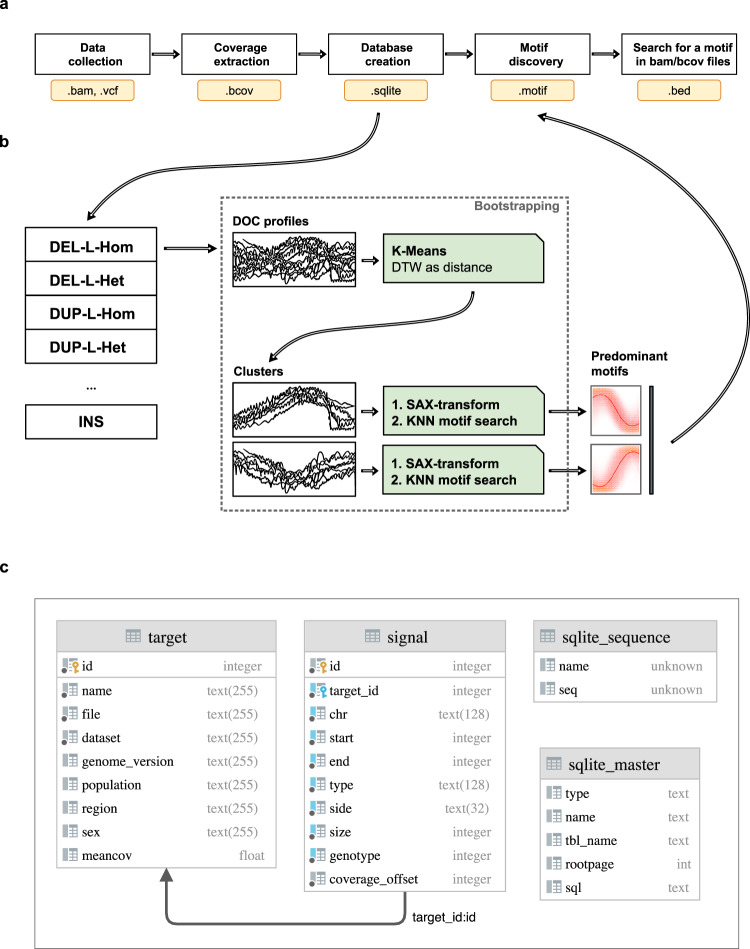
An overview of SWaveform framework. (**a**) SWaveform workflow. Data processing steps from raw annotated sequencing data to motifs and their detection in DOC profiles. (**b**) The bootstrapping procedure used for motif discovery from DOC profiles. (**c**) SWaveform database schema.

Here, for the first time we present a detailed catalogue of various waveforms and patterns observed in the sequence coverage signal associated with different types of SVs, as well as a toolkit for coverage data management and analytics. SWaveform provides easy access to approximately 7 M DOC signal profiles extracted from 911 human sequencing samples generated by the Human Genome Diversity Project (HGDP)^6,32^. A portable database architecture and provided API facilitate easy and seamless on-premises deployment encompassing data processing routines on all levels i.e., from raw aligned data to visual representation of coverage profiles (shown in Fig. 1a). We also propose a new binary format to manage sequence coverage data. Finally, as motif discovery has been successfully applied throughout a large range of domains such as medicine, finance, robotics and DNA analyses we designed an algorithm for motif extraction from the coverage signal. Taken together, SWaveform will be instrumental for in-depth studies of signal properties with an extensive body of dedicated algorithms commonly used in the signal processing domain for feature extraction, pattern discovery and anomaly detection. In addition, a collection of signals and patterns could facilitate the development of strategies for filtration of SVs detected by various callers and meta-callers. Also, SWaveform framework could be deployed locally to enable exploration of any sequencing data in a clinical or research context. Overall, the developed catalogue and accompanying toolkit form an indispensable resource that will facilitate development and honing of computational tools for discovery of specific genomic rearrangement events. It is expected that SWaveform will be of immense value to the machine learning and biomedical communities.

## Methods

### Data management

We used HGDP sequencing data^32^ which includes 911 whole-genome sequenced human samples in CRAM format^33^ with an average depth of coverage of about 30x. Aligned sequencing data was downloaded from the International Genome Sample Resource ftp site (http://ftp.1000genomes.ebi.ac.uk/vol1/ftp/data_collections/HGDP/data/). Structural variation data generated by the consortium contains annotated breakpoints for the following types of events: CNV gain and loss, insertions, inversions, deletions and duplications^6^, which amounts to ~15М DOC profiles. The corresponding set of structural variants (SV) encompassing 152,841 variants was obtained from the HGDP SV data repository (ftp://ngs.sanger.ac.uk/production/hgdp/hgdp_structural_variation/).

We developed a software suite to extract DOC profiles in a vicinity of SV breakpoints (Table 1). The default size of the region surrounding the breakpoint is imposed by the read length typically used in short-read sequencing experiments and comprises ±256 bp. Importantly, the parameter can be adjusted to accommodate for long-read sequencing protocols or to mitigate the consequences of imprecise breakpoint detection. SVs shorter than the window size, but exceeding 20 bp in length are labelled as “special” (i.e., spSV). Importantly, all SVs shorter than 20 bp are omitted. Furthermore, to speed up coverage data processing and optimise storage we introduced a simple lossless binary format for recording of coverage values (BCOV). The format was purposely developed to ensure fast and efficient programmatic access to the DOC data, which is encoded as follows. For each position on a chromosome a numeric value corresponding to the read coverage depth is stored in two bytes, saved in a binary file in a sequential order. Thus, the maximal supported coverage value is bounded by 2^16^. If the coverage exceeds the limit, the value is capped to the maximum of 65 536 reads. The size of an average BCOV file generated for the human genome amounts to about 5.5 Gb.

**Table 1:** Total number of DOC profiles in SWaveform database.

Breakpoint type/SV class	CNV gain	CNV loss	DEL	DUP	INS	INV
Left (L)	467, 686	652, 892	1, 211, 445	31, 042	0	35, 469
Right (R)	467, 716	643, 231	1, 211, 413	31, 042	0	35, 469
Break Point (BP)	0	0	0	0	1, 087, 106	0
spSV	0	0	1, 263, 414	46, 305	0	2, 187
Total	935, 402	1, 296, 123	3, 686, 272	108, 389	1, 087, 106	73, 125

The imbalance of the number of breakpoints (left and right) for several SV types (i.e., deletion) is caused by zero coverage values at the beginning/end of a variant.

The genomic data from CRAM files was processed with mosdepth tool^34^ to extract a numeric value reflecting sequencing read coverage depth for each genome position and converted it to BCOV. The mosdepth program is run with the default set of parameters to exclude reads characterised with a combination of bitwise FLAGs 1796. In essence, this results in a removal of the following read categories: segment unmapped, secondary alignment, not passing QC, PCR or optical duplicate. Next, the breakpoint coordinates of copy number variants, and of the following SVs, namely deletions, insertions, inversions, duplications were obtained from the corresponding VCF files. We further filtered VCF records to include only those variants distinguished with a PASS flag (i.e., Manta FT flag). Finally, the extracted profiles, breakpoint loci and sequencing samples metadata (7, 314, 329 entries in total) were stored in a relational database (SQLite) to facilitate data search, retrieval and visualization (see Data Records section and Fig. 1a,c).

### Motif discovery

A profound variability of waveforms associated with different classes of SVs has long impeded the reliability and reproducibility of the discovery algorithms. We, therefore, sought to identify repeated patterns found within DOC profiles (i.e., motifs) and characterise conserved structures in the signal.

Briefly, the procedure for motif discovery encompasses the following steps (Fig. 1b). First, the optimal number of representative clusters containing somewhat similar DOC signals in terms of shape of associated waveforms within the annotated SV classes is estimated. This step is run only once for every combination of SV type/breakpoint (i.e., left or right, if applicable). In the second step, the estimated number is used to cluster DOC profiles intrinsic to each of the aforementioned combinations. Next, to identify and rank motifs within each cluster we use K-nearest neighbour approach. Due to the large volume of data the latter step is run repeatedly on bootstrap samples from the original data. Finally, the motif groups emerging from each of the bootstraps are iteratively merged to pinpoint the most predominant one for each of the clusters. The details of every step of the procedure are outlined in the paragraphs below.

Although structural variation has been in the spotlight of genomic research in the last decade, the multiformity and diversity of signal profiles attributed to specific types of SVs have never been properly characterised. Furthermore, as structural variation data produced by the HGDP is not curated, it is highly likely that an a priori unknown number of false calls is present in the data set. To identify predominant waveforms characteristic to annotated SVs in the HGDP data the coverage profiles attributed to specific classes of SVs were compressed, normalised and clustered with dynamic time warping (dtw) distance^35,36^ as discussed in the Technical Validation section. The numerical experiments involving silhouette index and bootstrap resampling (80 runs, without replacement) used to estimate the optimal number of clusters associated with each SV type have demonstrated that the data partitioning into more than two subsets is not justified (see Supplemental Figs. 1,2). In the case that the most representative cluster (i.e., containing more than 66% of DOC profiles) can be identified, the motif discovery is restricted to it. Alternatively, the motif discovery is performed in both clusters. The latter scenario is likely to encompass those instances where the performance of the SV discovery algorithms is questionable and, consequently, the detected breakpoints are ambiguous. This particularly applies to CNV gains as discussed below.

**Fig. 2:**
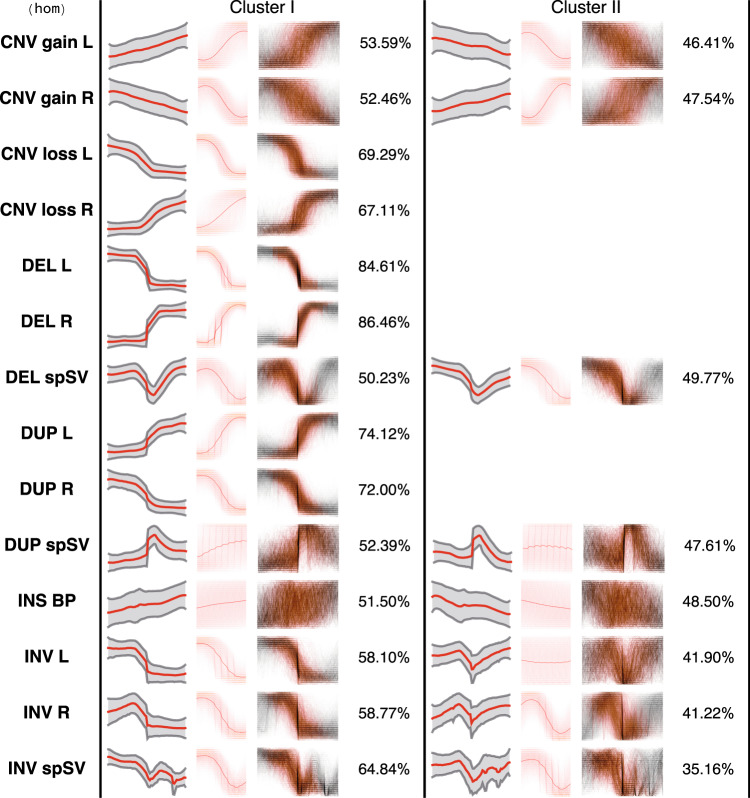
Signal profiles and motifs identified for specific combinations of SV classes/breakpoint types in the case of homozygous events. Each column in a table contains (from left to right): compressed, normalised DOC profile, where red line shows signal mean and grey shading corresponds to standard deviation; the predominant motif identified for a given SV/breakpoint type (see also Fig. 5); the SAX-transformed DOC profiles (thin grey lines) with the respective motifs (orange) projected onto them; percentage of profiles pertaining to a cluster. Cluster I and Cluster II are groups generated with K-Means method. Cluster II data is not shown if it covers less than 1/3 of DOC profiles.

**Fig. 3:**
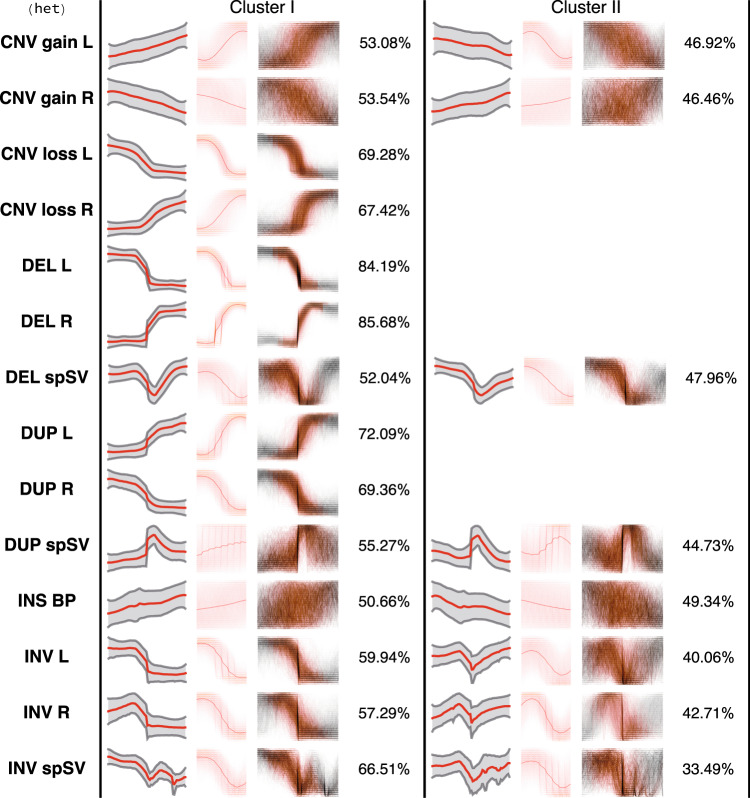
Signal profiles and motifs identified for specific combinations of SV classes/breakpoint types in the case of heterozygous events. Each column in a table contains (from left to right): compressed, normalised DOC profile, where red line shows signal mean and grey shading corresponds to standard deviation; the predominant motif identified for a given SV/breakpoint type (see also Fig. 5); the SAX-transformed DOC profiles (thin grey lines) with the respective motifs (orange) projected onto them; percentage of profiles pertaining to a cluster. Cluster I and Cluster II are groups generated with K-Means method. Cluster II data is not shown if it covers less than 1/3 of DOC profiles.

The motif discovery poses a significant computational challenge, as the total number of DOC profiles in the HGDP dataset amounts to ~7 M and the extracted profile length is 512 bp. We were, therefore, impelled to carry out the motif search in the dataset chunks associated with each type of SV, genotype and a corresponding breakpoint (i.e., left or right). The bootstrapping encompasses 360 subsets comprising 960 signal profiles for every SV/breakpoint/genotype combination. Thus, for every data subset compressed DOC profiles were clustered with K-Means algorithm (dtw distance) into two clusters (as justified in the above paragraph) to reveal predominant waveforms present in the data (see Fig. 1b). Due to combined imperfections of both read alignment and SV discovery algorithms the DOC profiles in the vicinity of a breakpoint are highly variable in shape and form, meaning that the signal can be either stretched or shifted. To account for variability, we apply SAX (Symbolic Aggregate approXimation) transformation^37^ to the signal using an alphabet of 24 symbols. Next, the overlapping sliding window-based segmentation (32 data points) was applied to the SAX-transformed signal. Finally, to discover the most significant motifs from the profiles, the resulting segments are fed into the modified *KNN_Search* algorithm^38^ which partitions them into similarity groups. Importantly, the *KNN_Search* algorithm was modified to facilitate efficient motif discovery (as discussed below in the Technical Validation section). The *KNN_Search* method yields a ranked list of similarity groups, characteristic for a given cluster. Тhe ranking reflects the group’s prominence. Finally, the motifs generated as a result of the bootstrapping are iteratively merged (using SAX distance-based thresholding) and averaged to reveal the most predominant one for each cluster (Figs. 2,3). To get a full understanding of the computational approach adopted, please refer to the source code in the Code Availability section.

The pattern in itself is an ample source of information on aberrations in the signal, that could arguably be used to draw valuable conclusions on the performance of the existing algorithms for SV discovery and on the waveforms characteristic to various types of SVs.

In particular, from our findings it follows that regardless of the genotype, the breakpoints corresponding to copy number gains are much harder to localise with precision, as the patterns associated with their SV breakpoints are blurred and exhibit gradual increase in signal intensity as compared to clear step-wise pattern observed in the case of duplications. Strangely, in structural variants annotated as CNV gains, irrespective of the beginning or end of the interval (i.e., left or right breakpoint) and genotype, two motifs with opposite trends in the coverage signal are observed (see Figs. 2,3). Moreover, each of these patterns is supported by relatively similar proportions of DOC profiles. Considering these observations, we may hypothesise that segmentation-based approach to boundary determination and possibly varying signal amplitude at the variant start (or end) locus confound CNV discovery software and result in ambiguous boundary attribute (e.g., left or right) of a variant.

Besides that, the motif discovery did not produce any convincing result in the event of insertion, which may indicate that the distortion on the DOC signal in the vicinity of the breakpoint do not go beyond superficial alterations (Figs. 2,3). Interestingly, we have detected two motifs coupled to breakpoints related to both hetero- and homozygous inversions. In fact, these clusters describe signal behaviour at the inversion boundaries (Supplemental Fig. 4), although admittedly the motif is less pronounced for the left boundary of the homozygous inversion. The latter is likely to be a consequence of a relatively small size of the data, as the number of homozygous inversion profiles included into analyses amounts to 11065 entries. Leaving aside the genotype data triples the number of profiles and allows for generation of a distinct SAX model (see Supplemental Fig. 4).

An exploratory analysis of motifs generated with spSVs demonstrate that typically the method is capable of capturing the signal around the breakpoint. As expected, the varying length of the variant downstream of the breakpoint clearly impacts the ability for recapitulation of the signal shape.

On the whole, in the case of both homozygous and heterozygous variants the best motifs are detected for the following classes of events: duplications, deletions, CNV loss and, possibly, inversions. It is quite within reason to suggest, that this result is a consequence of at least two factors, namely, the precision in discovery of breakpoints associated with the respective variants, as well as the distinct manifestation of the related waveforms.

The resulting motifs in SAX format, stored in the SWaveform database may have an important utility for a) development of novel improved approaches for breakpoint detection, and b) for visualisation of repeated patterns in the DOC signal.

## Data Records

Data presented in this work can be accessed directly at Zenodo repository^39–41^ as an archive in ZIP format, which includes SQLite dump, DOC signal profiles in BCOV format and the accompanying metadata in various formats. The database schema is presented in Fig. 1c and on the SWaveform website at swaveform.compbio.ru/description.

## Technical Validation

In this study various approaches were applied to validate reliability, integrity and quality of the raw and transformed data, as well as data processing.

The HGDP provides high quality data processed in accordance with SOPs as described in Almarri *et al*.^6^. The DOC values were extracted from the CRAM files and converted into lossless BCOV format. The breakpoint coordinates of the structural variants characterised by the aforementioned consortium were extracted from the provided VCF files and filtered to allow variants annotated with the PASS flag. The DOC profiles in the 512 bp neighbourhood centred on the filtered breakpoint were then extracted for samples with homo-/heterozygous genotype of the variant.

### Signal compression and clustering

In each bootstrap run the database was subsetted to select a 100 random signal profiles associated with a specific type of SV. To speed up the clustering procedure, signal profiles were compressed using average pooling in windows of 8 base pairs long. The compressed profiles were further normalised (i.e., scaled to zero mean and unit variance within a sequenced sample) and clustered with K-Means algorithm (as implemented in tslearn package^42^) using two different randomly selected seeds (e.g., cluster sets C0 and C1). Concurrently, the same group of signal profiles was compressed and clustered using the same seed as in C0 resulting in a cluster set C2. A percentage of profiles that retained their cluster association regardless of seed between cluster sets C0 and C1, as well as between C0 and C2 was computed.

Clustering procedures using both compressed and uncompressed signal profiles were repeated 80 times to generate distributions showing clustering consistency. The resulting distributions of profiles that retained their cluster association expressed as percentage from the overall number of profiles were compared using Kolmogorov-Smirnov one-sided two-sample test, as shown in Supplemental Fig. 3. Our numerical experiments clearly demonstrate that signal compression produces much less effect on cluster consistency as opposed to the seed selection, indicating that signal compression impact on clustering results is minor.

### Modified *KNN_Search* algorithm

**Fig. 4:**
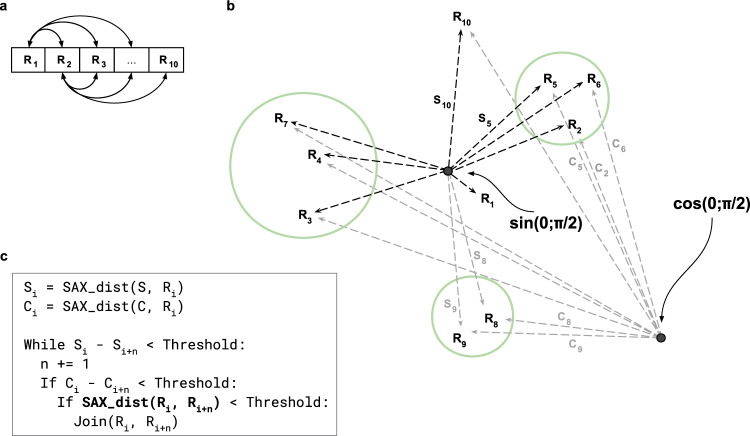
An overview of the modified *KNN_Search* algorithm. (**a**) Iteration order through the SAX-transformed DOC profiles in 1D space. (**b**) Clustering of DOC profiles (*R*_*i*_) in SAX space. *S*_*i*_ and *C*_*i*_ represent SAX distances between *i*_*th*_ DOC profile and sine and cosine reference points, correspondingly. (**c**) Outline of the modified *KNN_Search* algorithm.

The *KNN_Search* method published by Zaher Al Aghbari^38^ adopts heuristics approach to guide motif search and to identify stable patterns in the signal. *KNN_Search* rests upon the linearization concept i.e., it is reasoned that when two subsequences are close in the (multidimensional) SAX-space, these elements are also close in 1D space they are projected into. The linearization is achieved through selection of a reference node and subsequently ordering all other data points in accordance with their distances from it. The neighbourhood expansion is controlled through a threshold imposed on a distance between the reference point and a prospective group member. This allows for an efficient neighbour grouping in 1D space and reduces the search space for the time-consuming SAX distance calculations. To further improve computational efficiency of the method and scale down the SAX search space we introduced a second reference node and, consequently, an additional 1D space (see Fig. 4). Furthermore, instead of a randomly chosen reference node we opt to select two fixed distinct reference points, namely SAX-transformed sine and cosine functions on an interval of $\left[0,\frac{\pi }{2}\right]$. The SAX distance within the prospective neighbourhood is computed, if and only if, two nodes are close in each of the one-dimensional spaces. Thus, the optimization is achieved through narrowing down the search space in which SAX distance estimation is performed.

## Usage Notes

**Fig. 5:**
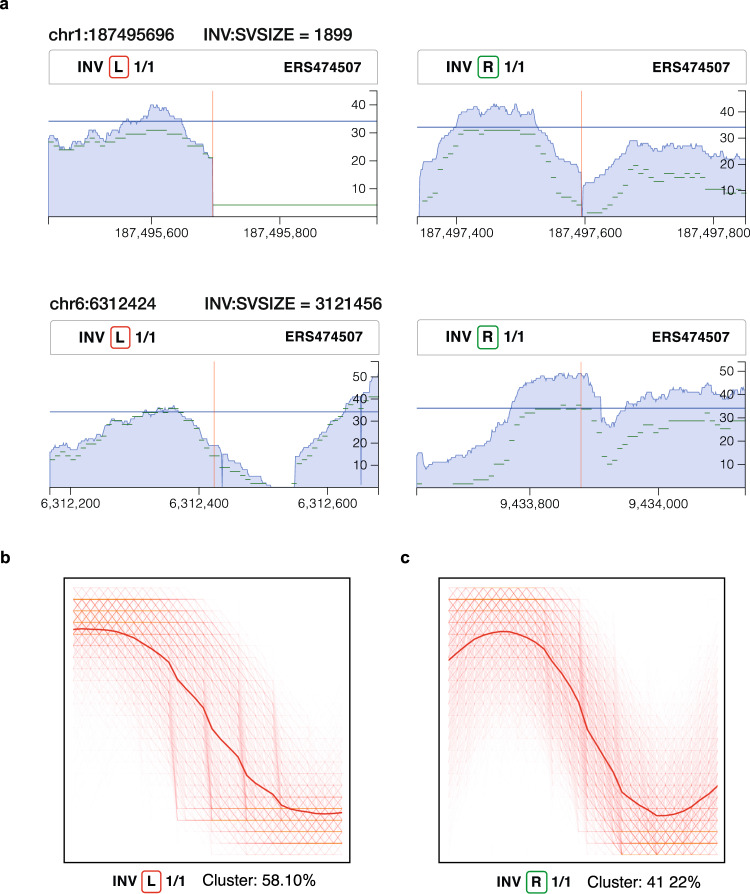
DOC profile examples corresponding to left and right breakpoints of two inversions annotated by the HGDP consortium and the associated SAX representation of predominant motifs. (**a**) The DOC profiles for two inversions. The HGDP sample id, the type of a structural variant, the genotype and the breakpoint type (i.e., left or right) are indicated above each DOC profile. The x -axis represents the depth of coverage of every single nucleotide position in respect to the reference sequence and the y-axis is the genomic position. Vertical red line shows breakpoint coordinate as reported by the HGDP. Mean coverage for the whole sample is displayed in horizontal solid blue line. Dashed green lines correspond to the SAX-transformed DOC profiles. (**b**) SAX representation of a motif associated with the 5′ breakpoint. (**c**) SAX representation of a motif associated with the 3′ breakpoint. Thin red lines show SAX representation of signal profiles generated as a result of *KNN_Search*, bootstrapping and merging i.e., those corresponding to the most predominant similarity group. The thick red line shows the predominant motif.

SWaveform resource can be accessed through graphical user interface (GUI) on swaveform.compbio.ru. The interface provides the ability to visualise profiles, search by genomic coordinates, and filter by ethnic group provided by the HGDP, SV class, genotype and breakpoint type. The interface also provides chromosome browser capability. The user part of the interface (front-end) is implemented using React.js and D3.js tools, while the server part (back-end) is written in Python with the flask framework. Examples of signal profile visualisation both for individual samples and for averaged profiles corresponding to one or another type of structural variation are shown in Fig. 5. In addition, the application programming interface (API) to the database was developed, allowing direct access to the data from the user’s programs by means of Python or PHP.

The predominant motifs associated with a given combination of SV type/breakpoint are provided as a SAX transformation, which enables scanning of DOC profiles encoded in BCOV format for possible anomalies and aberrations. This solution is implemented in C and Python and is available as a part of the software suite accompanying the resource.

To showcase the resource in action we provide two Snakemake workflows (please see Code availability section for details). The first one encompasses all steps required for resource deployment from user’s data. The workflow generates all the files necessary to set up a local database of DOC profiles and extracts coverage signals in the vicinity of breakpoints to build a set of predominant motifs associated with a given combination of SV type/breakpoint. The second workflow is a prototype to demonstrate a practical implementation of a simple pattern search in the data to facilitate anomaly detection in the DOC signal. Both workflows use moderate-sized datasets available on Zenodo^40,41^.

The database population workflow is shown in Fig. 1a.

## Supplementary information

Supplementary Information

## Data Availability

A software suite accompanying the resource is available on https://github.com/latur/SWaveform. The repository contains scripts for a) database and GUI deployment on the SQLite platform and b) a toolkit for DOC profile and SV data processing and management. The toolkit contains scripts for generation of DOC profiles corresponding to breakpoint loci from alignment files (SAM, BAM or CRAM format) and annotated VCF files, as well as DOC profile conversion into BCOV format. In addition, we provide tools for profile clustering, motif discovery and a script for subsequent motif detection in DOC profiles.
