# Supplementary material for: A collection of read depth profiles at structural variant breakpoints
Source: Sci Data. 2023 Apr 6;10:186. doi: 10.1038/s41597-023-02076-4 (PMC10079824; doi:10.1038/s41597-023-02076-4)
Supplement: Supplementary file 1 — Supplementary Information [file 41597_2023_2076_MOESM1_ESM.pdf]

Table of Contents

Supplementary Figure 1. Silhouette index distributions across different heterozygous SV/breakpoint types.

Supplementary Figure 2. Silhouette index distributions across different homozygous SV/breakpoint types.

Supplementary Figure 3. The influence of DOC profile compression on K-Means clustering (K=2) result.

Supplementary Figure 4. Signal profiles and motifs identified for both heterozygous and homozygous inversions.

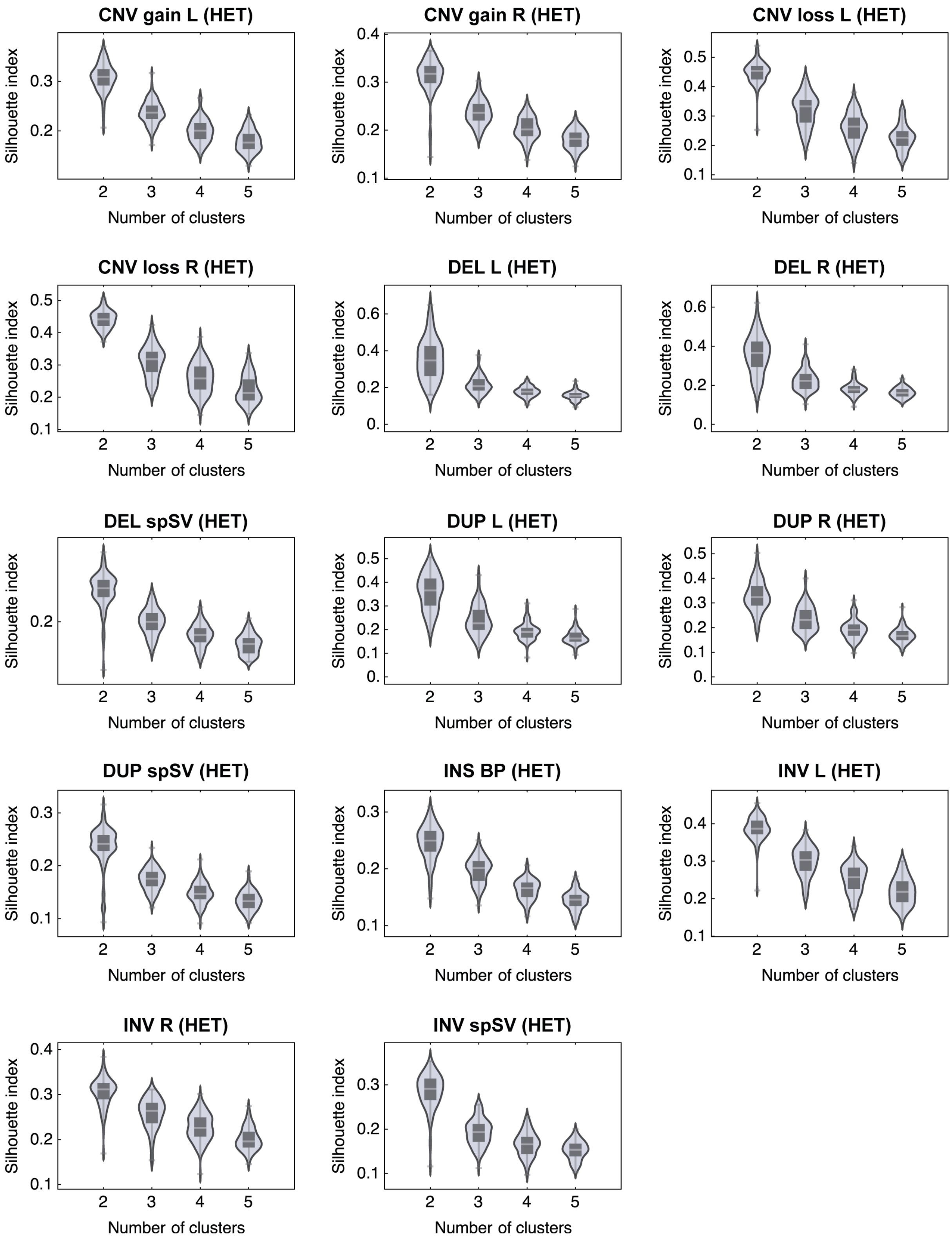

Supplemental Figure 1. Silhouette index distributions across different heterozygous SV/breakpoint types. Each chart corresponds to a specific combination of structural variant class and breakpoint type. The hybrid of a box plot and a kernel density plot (violin) shows summary statistics and a corresponding distribution of silhouette indices calculated for different number of data partitions obtained as a result of bootstrapping procedure and K-Means clustering of compressed, normalized DOC profiles.

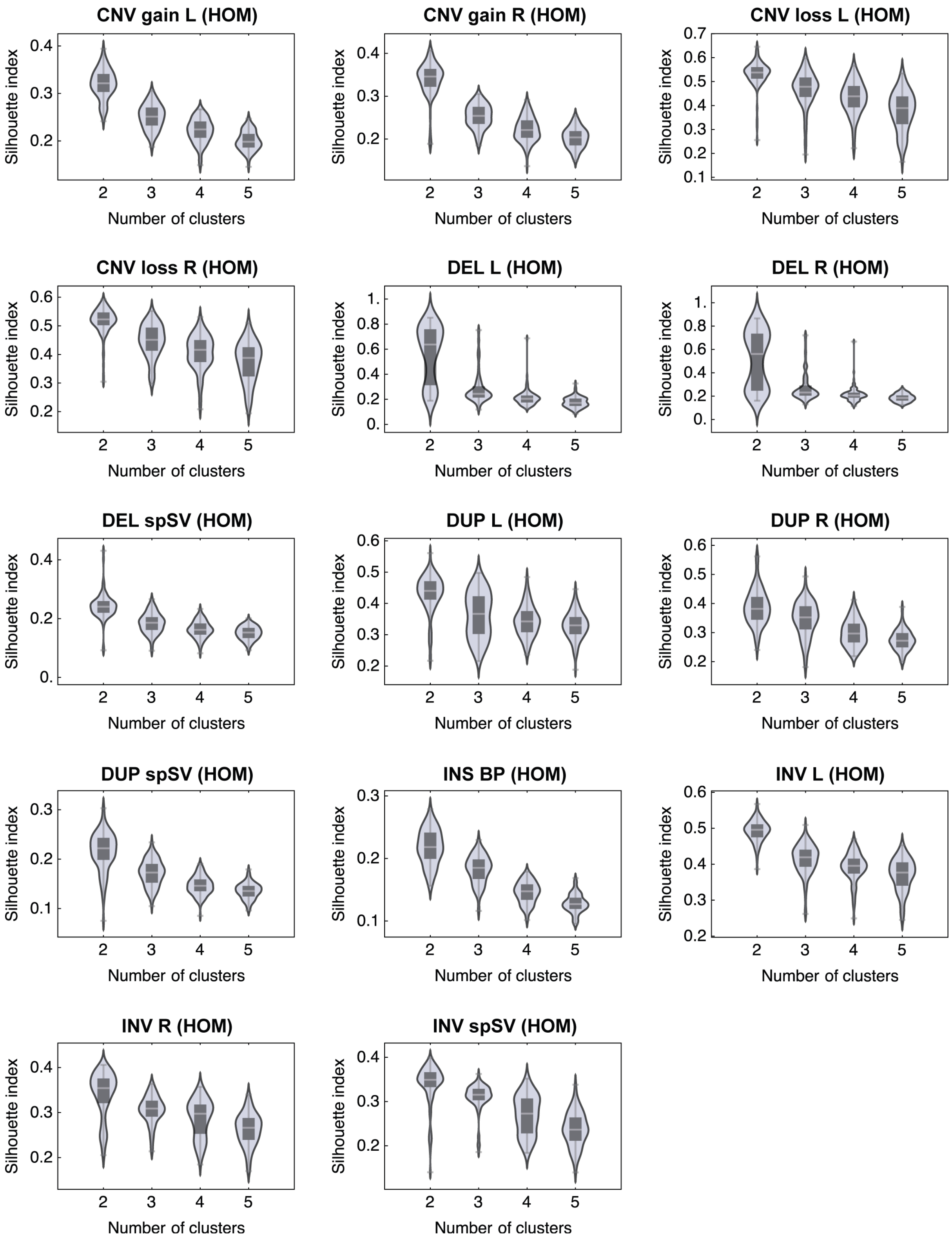

Supplemental Figure 2. Silhouette index distributions across different homozygous SV/breakpoint types. Each chart corresponds to a specific combination of structural variant class and breakpoint type. The hybrid of a box plot and a kernel density plot (violin) shows summary statistics and a corresponding distribution of silhouette indices calculated for different number of data partitions obtained as a result of bootstrapping procedure and K-Means clustering of compressed, normalized DOC profiles.

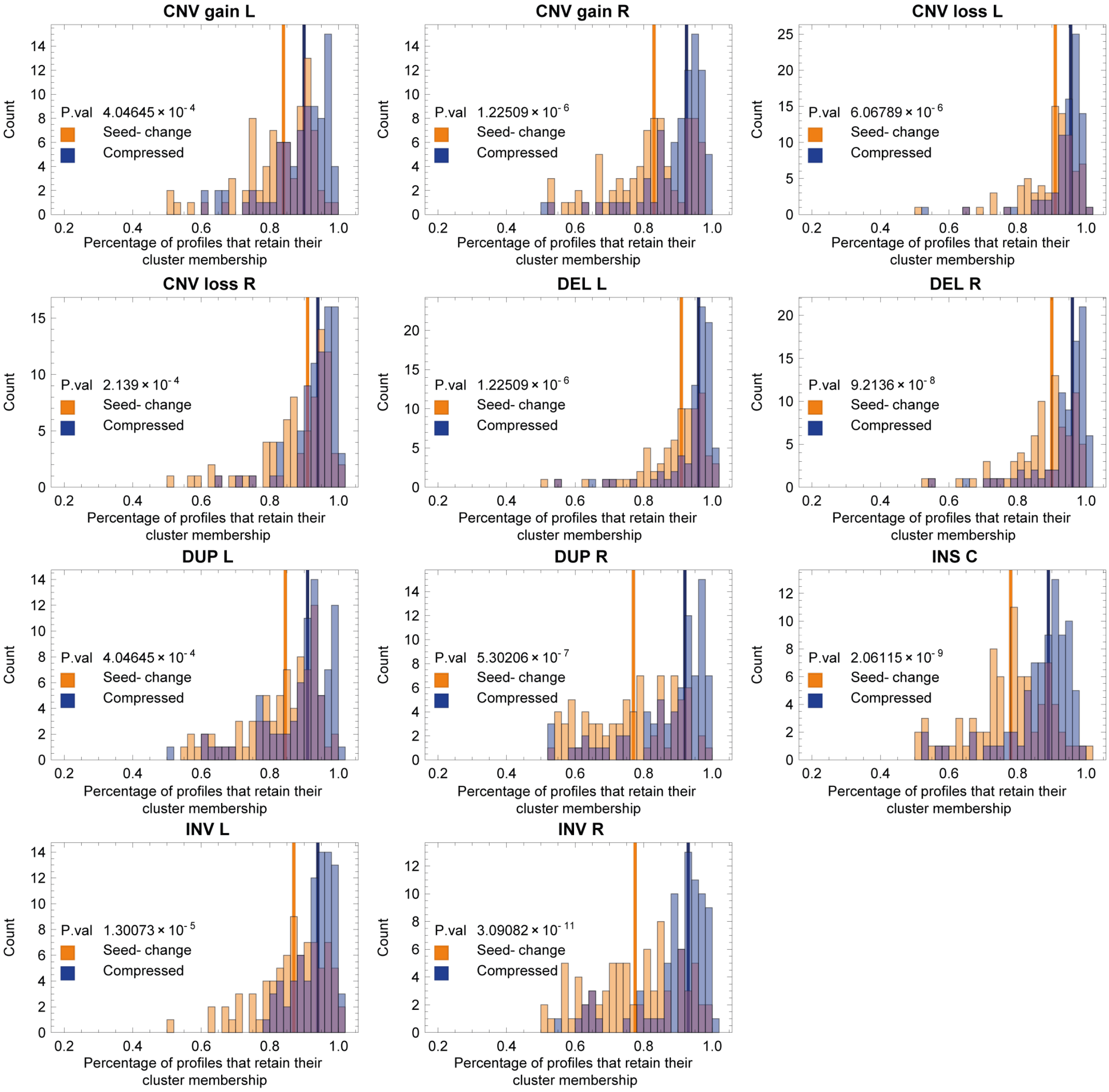

Supplemental Figure 3. The influence of DOC profile compression on K-Means clustering (K=2) result. Each chart corresponds to a specific combination of structural variant class and of a breakpoint type. The histograms present the results of a bootstrapping procedure designed to evaluate cluster association consistency produced with K-Means algorithm run on either compressed DOC profiles (blue histogram, same seed) or unperturbed DOC profiles (orange histogram). In the latter case we assessed alterations in cluster composition caused by random seed change. The blue and orange vertical lines correspond to respective distribution means.

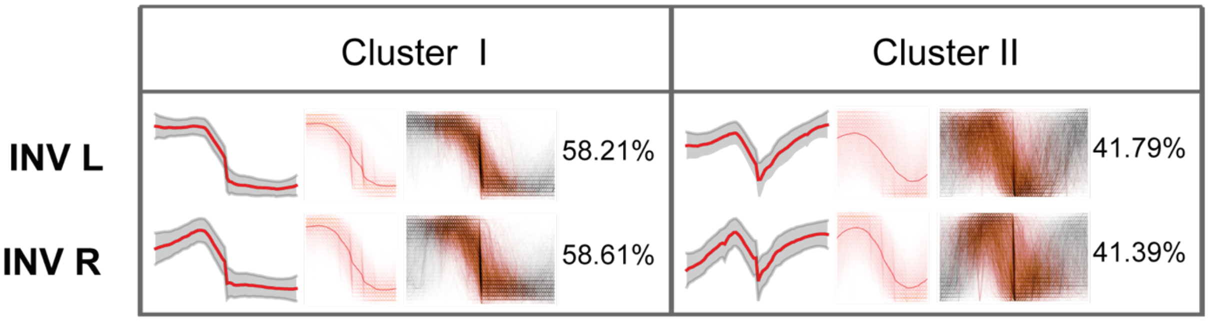

Supplemental Figure 4. Signal profiles and motifs identified for both heterozygous and homozygous inversions. *Each column in a table contains (from left to right): compressed, normalised DOC profile, where red line shows signal mean and grey shading corresponds to standard deviation; the predominant motif identified for a given breakpoint type (i.e., left or right); the SAX-transformed DOC profiles (thin grey lines) with the respective motifs (orange) projected onto them; percentage of profiles pertaining to a cluster. Cluster I and Cluster II are groups generated with K-Means method.*
